# Activation of 5-HT_2_ Receptors Reduces Inflammation in Vascular Tissue and Cholesterol Levels in High-Fat Diet-Fed Apolipoprotein E Knockout Mice

**DOI:** 10.1038/s41598-019-49987-0

**Published:** 2019-09-17

**Authors:** Thomas W. Flanagan, Melaine N. Sebastian, Diana M. Battaglia, Timothy P. Foster, Emeline L. Maillet, Charles D. Nichols

**Affiliations:** 1 Department of Pharmacology and Experimental Therapeutics Louisiana State University Health Sciences Center 1901 Perdido St, New Orleans, LA 70112 USA; 2 Department of Microbiology, Immunology, and Parasitology Louisiana State University Health Sciences Center 1901 Perdido St, New Orleans, LA 70112 USA; 3 Eleusis Benefit Corporation 11 East 44th St., Suite 104, New York, NY 10017 USA

**Keywords:** Vascular diseases, Cardiology

## Abstract

Coronary artery disease (CAD) is a progressive cardiovascular syndrome characterized by cholesterol-induced focal arterial lesions that impair oxygen delivery to the heart. As both innate and adaptive immune cells play critical roles in the formation and progression of arterial plaques and endothelial cell dysfunction, CAD is commonly viewed as a chronic inflammatory disorder. Our lab has previously discovered that 5-HT_2A_ receptor activation with the 5-HT_2_ receptor selective agonist (*R*)-2,5-dimethoxy-4-iodoamphetamine [(*R*)-DOI] has potent anti-inflammatory activity in both cell culture and whole animal models. Here we have examined the putative therapeutic effects of (*R*)-DOI in the ApoE^−/−^ high fat model of cardiovascular disease. Subcutaneously implanted osmotic minipumps were used to infuse sustained low rates (0.15 μg / hr) of (*R*)-DOI∙HCl to mice fed a high-fat “Western” diet. (*R*)-DOI treated mice had significant reductions in expression levels of mRNA for inflammatory markers like *Il*6 in vascular tissue, normalized glucose homeostasis, and reduced circulating cholesterol levels. As cardiovascular disease is a leading cause of death both globally and in the Western world, activation of 5-HT_2A_ receptors at sub-behavioral levels may represent a new strategy to treat inflammation-based cardiovascular disease.

## Introduction

Cardiovascular disease and its clinical manifestations are the leading cause of morbidity and mortality worldwide, accounting for more than 17.3 million deaths per year^1,2^. For atherosclerotic cardiovascular disease (ASCVD), despite significant advances in patient treatment recurrent cardiac events, cardiovascular death and treatment costs remain high^3,4^. While approaches such as statin therapy lower low-density lipoprotein (LDL) cholesterol levels and reduce cardiovascular events^5,6^, many patients need further LDL cholesterol lowering and still remain at risk for adverse cardiovascular events^7^. Furthermore, while statins do possess anti-inflammatory properties^8^, in a subset of individuals there are negative pleiotropic effects, namely myotoxicity^9^. While management options for statin intolerant patients exist^10^, alternative therapies to combat ASCVD are continuously being developed^7,11,12^.

One potential alternative therapeutic route to treat cardiovascular disease is modulation of the serotonin system^13^. Serotonin [5-hydroxytryptamine (5-HT)] is a small, ubiquitous monoamine found in nearly all eukaryotes that functions as a neurotransmitter in the central nervous system (CNS), and modulates cardiovascular, gastrointestinal, developmental, and endocrine function in the periphery^14^. In mammals serotonin exerts its effects through activity at seven different families of receptors comprised of fourteen distinct subtypes^14^. With the notable exception of the 5-HT_3_ receptor, which is a ligand-gated ion channel, all are seven transmembrane-spanning G-protein-coupled receptors (GPCRs). One of the subtypes, the 5-HT_2A_ receptor, plays pivotal roles in mediating complex cognitive behaviors like working memory in the CNS^14,15^ and physiological processes like vasoconstriction in the periphery^13,16^. The most widely expressed mammalian receptor subtype, the 5-HT_2A_ receptor^14,17^, has been detected in nearly every tissue type examined including endothelial, muscle, endocrine, and immune^18–21^. Of note, the 5-HT_2A_ receptor is the target of classic serotonergic hallucinogens, or psychedelics^22^, which may in part explain why the majority of scientific research on 5-HT_2A_ receptors has focused on deciphering its role in the CNS^17^.

Only recently have the peripheral roles of 5-HT_2A_ receptors begun to be identified^17^. With respect to cardiovascular function, 5-HT_2A_ receptors in vascular smooth muscle and endothelial cells, as well as in cardiomyocytes are believed to partially mediate cellular proliferation and vasoconstriction^16,23^. We previously found that activation of 5-HT_2A_ receptors in primary aortic smooth muscle cells inhibit tumor necrosis factor (TNF)-α-mediated inflammation with extraordinary potency^24^. Administration of the 5-HT_2_ receptor selective agonist (*R*)-DOI to mice prior to TNF-α treatment to mice prevents expression of cell adhesion markers (intracellular adhesion molecule-1 and vascular adhesion molecule-1), cytokines (IL-6 and IL-1b), and chemokines (monocyte chemotactic protein-1 and chemokine (C-X3-C motif) ligand 1 *Cx3cl* 1)^24^. (*R*)-DOI was also able to completely block TNF-α-induced circulating IL-6 levels^24^. The highest dose of (*R*)-DOI used in that study, 0.3 mg/kg, is the lowest behaviorally active dose in mice^25^, indicating that that the therapeutic effects of (*R*)-DOI-mediated 5-HT_2A_ receptor activation may be achieved at sub-behavioral levels.

Because the 5-HT_2A_ receptor has been detected in cardiovascular tissues and 5-HT_2A_ receptor activation has potent anti-inflammatory effects in these tissues, we hypothesized that psychedelics may be a novel therapeutic to treat cardiovascular disease^13^. To investigate this, we used subcutaneous osmotic minipumps to deliver (*R*)-DOI at sustained low infusion levels to apolipoprotein E (ApoE) knockout mice (ApoE^−/−^) fed a high-fat diet (HFD). ApoE mediates lipoprotein clearance and cholesterol transport^26,27^; thus, its deficiency results in low HDL levels and lipoprotein accumulation. ApoE^−/−^ mice fed a HFD have excessive accumulations of total cholesterol (TC) and low-density lipoprotein cholesterol (LDL-C) in their vascular walls^28^. Accordingly, these mice are used as a model to study the development of cardiovascular disease^29^. We observed that a HFD increased circulating levels of total cholesterol and TNF-α, increased inflammatory gene expression in the thoracic aorta, and impaired glucose tolerance in the animals. Low steady-state drug plasma levels of (*R*)-DOI (~0.5 ng/ml) were associated with reduced *Il*6, *vcam*1, and *mcp1* mRNA expression levels in aorta, significantly decreased total cholesterol, and restored glucose homeostasis.

## Materials and Methods

### Drug

(*R*)-DOI was provided by Dr. Bruce Blough (Research Triangle Institute, Research Triangle Park, NC) dissolved in sterile saline prior to osmotic pump filling.

### Animals

Young adult male *ApoE*^−/−^ mice in a C57BL/6 genetic background were purchased from The Jackson Laboratory (Bar Harbor, ME, USA) and used for experiments in their 10^th^ week of age (mean weight of mice ± SEM on the day of pump implantation 20.8 ± 0.2 g). All mice were maintained in the animal care facility at LSUHSC in ventilated cages in a temperature-controlled Specific Pathogen-Free room with 12 h dark/12 h light cycling; with the exception of 14 h fast period prior to blood sample collection for glucose tolerance testing (GTT; described below). All animals were allowed *ad libitum* access to food and water. All protocols were prepared in accordance with the Guide for the Care and Use of Laboratory animals and approved by the Institutional Animal Care and Use Committee at Louisiana State University Health Sciences Center.

### Diet and tissue collection

**Figure 1:**
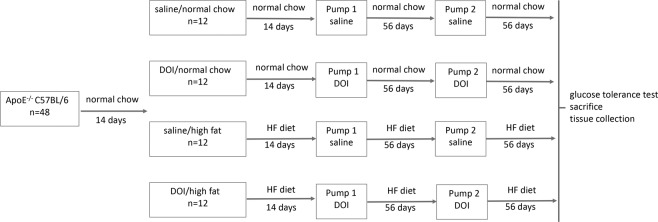
Experimental design and treatment schedule. Following 2 weeks of a normal chow diet, *ApoE*^−/−^ mice (n = 12) were fed a high-fat (HF; n = 12) diet. 2 weeks post diet change, mice were implanted with an osmotic mini-pump releasing either saline (saline/normal chow, saline/high fat) or (*R*)-DOI (DOI/normal chow, DOI/high fat). Mice were maintained for an additional 8 week before a second mini-pump was implanted into the opposing flank. 8 weeks post Pump 2 implantation a glucose tolerance test was performed followed by sacrifice and thoracic aorta dissection and blood collection.

Details of the experimental design are summarized in Fig. 1. Following a two week acclimatization period in which all mice were fed a regular chow diet (Teklad 7012; 5% fat, 19% protein, 5% fat; Harlan Teklad, Madison, WI,USA), animals were divided into 4 groups: *ApoE*^−/−^ mice fed a regular chow diet; mice to receive (*R*)-DOI fed a normal chow diet; *ApoE*^−/−^ mice fed a ‘Western’ high-fat (HF) diet (Teklad 97070; 21% fat by weight, 0.15% cholesterol); and mice to receive (*R*)-DOI fed a HF diet. Two weeks post HF diet initiation an Alzet mini-osmotic pump (Model 2006; lot no. 10310-13; mean fill volume 237.2 ± 3.0 μl, 0.15 ± 0.01 μl/hr infusion rate; calculated release of 3.6 ± 0.24 μl/24 hours for 65.9 (±0.6) days; DURECT Corp., Cupertino, CA, USA) preloaded with sterile saline (Baxter Healthcare Corp., Deerfield, IL) or (*R*)-DOI•HCl (2.4 mg/240 μl; 28 mM) dissolved in sterile saline were implanted subcutaneously in *ApoE*^−/−^ normal chow, (*R*)-DOI-normal chow, ApoE^−/−^HF, and (*R*)-DOI-HF mice to deliver a flow rate k_0_ of 1.5 μg/hr. For each surgery, mice were anesthetized with isoflurane (Piramal Healthcare, Andhra Pradesh, India) delivered via nosecone using a vaporizer system (SurgiVet Inc., WI, USA) with isoflurane at 2% in an oxygen flow of 1L/min. Implantation involved making a small incision in the skin on the left flank of each mouse and creating a small subcutaneous pocket using a halsted-mosquito hemostat (FST, Foster City, CA, USA). Pumps were inserted into the pocket of each mouse and the skin incision was closed using 4-0 silk sutures (Oasis Medical, Mettawa, IL, USA). The topical antibiotic Neosporin (Johnson & Johnson Consumer product, Inc., Skillman, NJ, USA) was used post-operatively to prevent infection at the implantation site. Eight weeks post implantation a second osmotic minipump prepared as described above with either sterile saline or drug was implanted subcutaneously into the opposing flank as described above. Animals were maintained an additional eight weeks (16 weeks total drug exposure) before euthanization via a single injection of ketamine/xylazine (60 and 3 mg/kg), thoracic cavity opening, cardiac puncture, exsanguination, and collection of blood. Serum was isolated by centrifuging blood in heparin coated tubes (BD, Billerica, MA, USA), which was flash frozen and stored at −80 °C. Whole aorta samples from the subclavical branch point to the iliac artery were dissected. The aortic arch was then dissected (aortic valve to first branch of aorta) and immediately snap frozen with 2-methylbutane (Sigma, St. Louis, MO, USA, Lot No. SHBJ9704, ≥99%) on dry ice, with storage at −80 °C until further use.

### Serum levels of (*R*)-DOI

Quantification of drug levels in plasma samples was performed at Pharmoptima LLC (Portage, MI) using optimized methods for (*R*)-DOI with a LLOQ of 0.1 ng/ml. The LC-MS/MS system used a Thermo Finnigan Quantum Ultra triple-quadrupole mass spectrometer coupled to a Thermo Finnigan Accela HPLC system (San Jose, CA). The HPLC system was equipped with a CTC Analytics HTC PAL autosampler (Zwingen Switzerland). Data acquisition and analysis was performed using Xcalibur V2.0.7, and LCquan V2.5.6 software on 11 samples. The overall mean steady-state trough concentration of plasma (*R*)-DOI (C_ss_) was 0.515 ± 0.20 ng/ml (mean ± SEM) (median of 0.385) with values ranging from <LLOQ to 1.29 ng/ml.

### Glucose tolerance test

Mice were placed in clean cages with no food but free access to water at ~7:00 p.m. After an overnight starvation (~12 hr; dark cycle), mice were weighed and a baseline blood sample was obtained by tail bleed. Mice were injected i.p. with glucose (1 g/kg body weight), and additional blood samples (~3 μl) were taken at the indicated time points for measurement of glucose concentration using an Accu-Check Mobile glucometer (Roche, Basel, Switzerland).

### Lipoprotein measurement

Serum lipids were determined enzymatically using commercial kits for total cholesterol and high density cholesterol (Total Cholesterol E-test and HDL Cholesterol E-test, respectively; Wako Pure Chemical Industries; Osaka, Japan) according to the manufacturer’s instructions.

### Quantitative real-time PCR

All nucleic acid was extracted and processed as described previously^24^. Briefly, total cellular RNA was isolated using TRIZOL reagent (Life Technologies, Carlsbad, CA, USA), per the manufacturer’s instruction. First-strand cDNA was generated using the ImProm-II cDNA synthesis kit (Promega, Madison, WI, USA), per the manufacturer’s instruction, with 500 ng total RNA per reaction. All primers for quantitative real-time PCR (qPCR) were synthesized by Integrated DNA Technologies, Inc. (Corralville, IA, USA), and designed to be compatible with probes from the Universal ProbeLibrary system using the Universal ProbeLibrary Assay Design Center (Roche Diagnostics, Indianapolis, IN, USA). Primer sequences and probes used in this study are listed in Table 1. Quantification of gene expression using a two-step cycling protocol was performed on a Roche LightCycler 480 Instrument II LC (Roche Diagnostics). Relative gene expression was calculated using the 2^−ΔΔCt^ method, with the levels of all targets from genes of interest normalized to internal *Gapdh* expression as determined using the Mouse Gapdh Gene Assay (Roche) in multiplex format.

**Table 1:** Gene expression analysis. Primer sequences and Universal Probe Library probe numbers used for q-RTPCR experiments to determine gene expression levels in aortic tissues.

Gene	Primer	Sequence (5′-3′)	Universal Probe Library (Roche) Probe no.
*Il6*	Sense	GCTACCAAACTGGATATAATCAGGA	6
	Anti-sense	CCAGGTAGCTATGGTACTCCAGAA	
*vacm1*	Sense	AACAACCGAATCCCCAACTT	83
	Anti-sense	TGATTGGGAGAGACAAAGCA	
*mcp-1*	Sense	GATCATCTTGCGGTGAATGAGT	62
	Anti-sense	CATCCACGTGTTGGCTCA	
*tnf*-*α*	Sense	TTGAGATCCATGCCGTTG	25
	Anti-sense	CTGTAGCCCACGTCGTAGC	
*gapdh*	Sense	AATCTCCACTTTGCCACTGC	GAPDH Assay Probe (Roche)
	Anti-sense	ATGGTGAAGGTCGGTGTGA	

### Cytometric bead assay

Serum from treatment groups was collected and processed as described above. A cytometric bead array was used to probe for the pro-inflammatory cytokines and chemokines using the Milliplex cytometric bead array kit (Millipore Sigma, Burlington MA Cat # MCYTOMAG-70K-PMX) according to the manufacturer’s instructions. The samples were run on a BioRad Bio-Plex 200 system and data was analyzed relative to a 5-parameter logistical standard curve for each corresponding cytokine/chemokine using Bio-Plex Manager 6.1.1.

### Statistical analysis

Statistical analysis was performed using GraphPad Prism v 7.0 software (GraphPad Software, La Jolla, CA, USA). All groups were compared by one-way analysis of variance (ANOVA), followed by Tukey post-test. Results are expressed as mean ± standard error of mean (SEM). *P* < *0*.*05* was considered to be significant.

## Results

### 5-HT_2A_ receptor activation via (*R*)-DOI does not impact food intake or body weight

**Figure 2:**
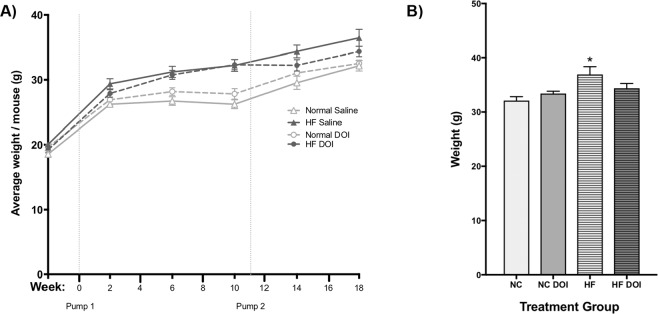
(*R*)-DOI does not impact weight gain or food intake. (**A**) Body weight was measured weekly for each animal following pump implantation and averaged over the course of treatment for each treatment group. (**B**) Body weight was measured each morning during the final week of treatment and averaged over a course of 7 days for each treatment group. *p < 0.05 one-way ANOVA with Tukey post-hoc test.

Early studies have indicated that several 5-HT_2A_ agonists like LSD have an anorexigenic effect^30^. Further, Lorcaserin, a 5-HT_2C_ preferential agonist with 5-HT_2A_ activity, has been shown to reduce body weight in obese men and women^31^, a property which is attributed to direct activation of 5-HT_2C_ receptors in the central nervous system (CNS)^32^. To test if sustained delivery of low-dose (*R*)-DOI had an appetite suppressing effect in our model, food intake was measured during the final week of experimentation. No significant decrease in food intake was observed between experimental groups given a high fat diet (data not shown). As expected, the mice fed a HF diet demonstrated significantly increased body weight (Fig. 2). HF fed mice implanted with (*R*)-DOI osmotic minipumps showed a trend for a very slight decrease in weight, but this was not significant (Fig. 2). Taken together these data indicate that sustained delivery of low-dose (*R*)-DOI results in drug levels that do not influence food intake or body weight.

### (*R*)-DOI increases glucose tolerance in ApoE^−/−^ mice fed a high fat diet

**Figure 3:**
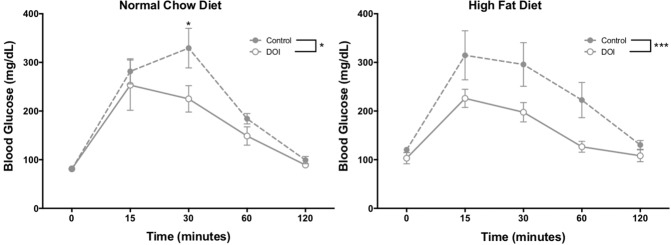
5-HT_2A_ receptor activation improves glucose tolerance in HF-diet and normal chow ApoE^−/−^ mice. Following sixteen weeks of a normal chow (**A**) or HF (**B**) diet glucose tolerance tests were performed for all treatment groups. Glucose tolerance was significantly improved for all (*R*)-DOI-treated animals. Data expressed as mean ± standard error. n = 9–10 for each treatment group. *p < 0.05.

A HF diet in mice has been shown to induce glucose intolerance^15,33^, a major predictor of metabolic and cardiovascular disease in humans^34^. To evaluate the impact of (*R*)-DOI on glucose sensitivity, we measured blood glucose concentrations in fasting mice after 16 weeks for treatment groups (Fig. 3). (*R*)-DOI improved glucose tolerance in both HF fed and normal chow fed treatment groups, indicating that 5-HT_2_ receptor activation can modulate the impaired glucose tolerance induced by a Western diet.

### 5-HT_2_ receptor activation lowers total and HDL cholesterol levels in Apoe^−/−^ mice

**Figure 4:**
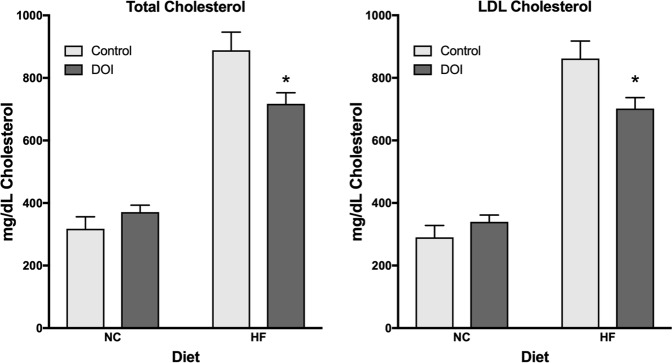
(*R*)-DOI lowers total cholesterol and LDL cholesterol in HF-diet fed mice. Serum was obtained from ApoE^−/−^ mice fed a normal chow or HF diet for 16 weeks. (*R*)-DOI decreased both total cholesterol (left) and HDL-cholesterol (right) in HF-diet fed mice. No significant effect was observed for normal chow-fed mice. Data represents the mean ± SEM. *p < 0.05; one-way ANOVA with Tukey post-hoc test.

ApoE^−/−^ mice fed a HF diet exhibited total cholesterol levels that were roughly three times higher than ApoE^−/−^ mice fed a normal chow diet (Fig. 4A). The presence of (*R*)-DOI modestly but significantly reduced total serum cholesterol levels. In a similar manner, HF diet elevated LDL cholesterol levels (Fig. 4B) was also moderately reduced in ApoE^−/−^ mice treated with (*R*)-DOI.

### (*R*)-DOI reduces the expression of proinflammatory marker genes in the aortic arch

**Figure 5:**
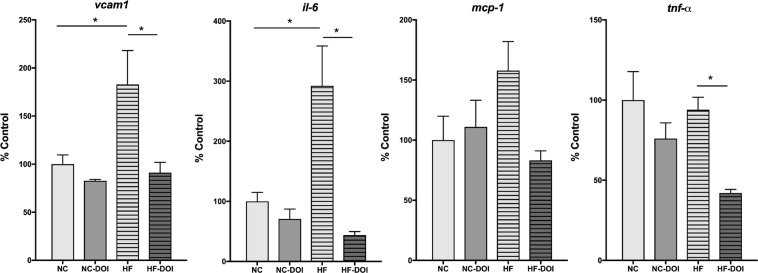
(*R*)-DOI decreases HF-diet induced pro-inflammatory gene expression in aortic tissue. Thoracic aorta were dissected and processed for RNA. qRT-PCR was performed to analyze the expression of pro-inflammatory genes. Levels of *Il*-6, *vcam*-1, and *tnf*-*α* we all elevated in HF-diet fed animals, and significantly reduced in (*R*)-DOI/HF diet animals. n = 5–6 animals per group; *p < 0.05 one-way ANOVA with Tukey post-hoc test.

Inflammation is strongly linked to the development of numerous cardiovascular disorders^35^. We have previously shown that (*R*)-DOI potently blocks TNF-α-induced mRNA expression of pro-inflammatory cell adhesion genes (v*cam*-*1*), cytokines (*Il*-*6*), and chemokines (*mcp*-*1*) in aortic tissue^24^. Here we wanted to examine proinflammatory gene expression in the context of HF diet-induced inflammation, and the effect of (*R*)-DOI on this expression. Thoracic aorta were dissected and the expression of several inflammation-related genes was evaluated (Fig. 5). mRNA for *Il*-*6*, *vcam1*, and *tnf*-*α* were all elevated in the HF fed group, indicating the presence of vascular inflammation. (*R*)-DOI treatment significantly decreased the expression of all three mRNAs. There was a trend for increased *mcp*-*1* expression in the HF fed animals that was prevented by (*R*)-DOI, however, these changes were nonsignificant.

### HF diet alters CXCL10 expression in the serum

**Figure 6:**
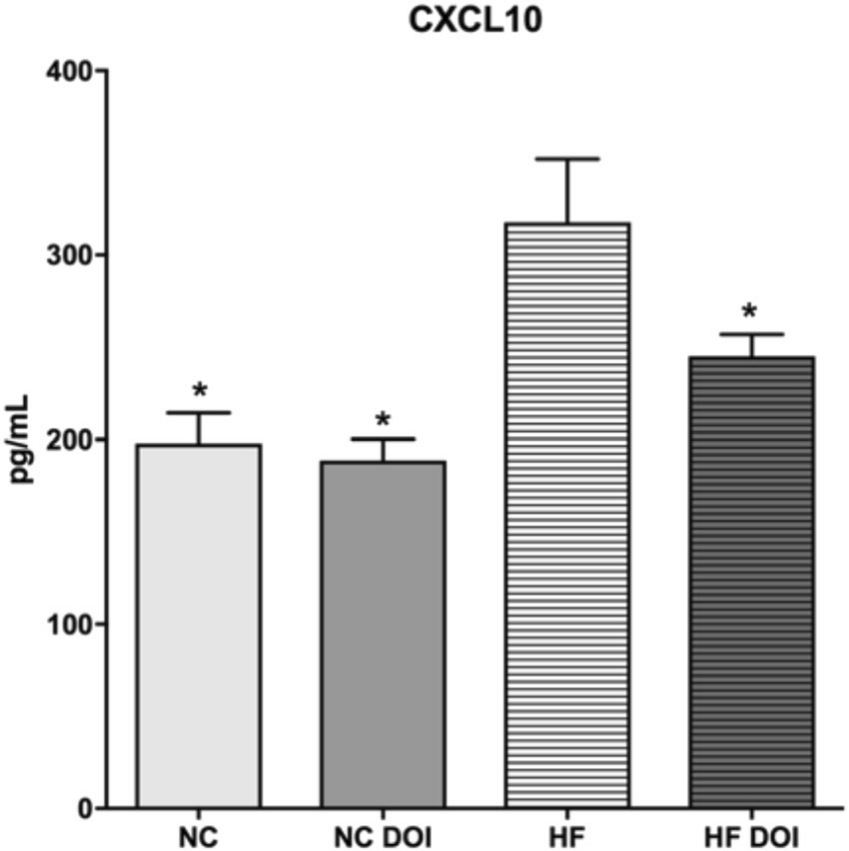
HF diet increases serum levels of the pro-inflammatory cytokine CXCL10 in ApoE^−/−^ mice. Serum was obtained via cardiac puncture of ApoE^−/−^ mice fed a normal chow or HF diet for 16 weeks and subjected to cytometric bead array analyses for CXCL10. HF diet elevated serum levels of the pro-inflammatory cytokine CXCL10 relative to HF diet DOI mice. n = 6–8 animals per group; ^#^p < 0.05 vs HF; one-way ANOVA with Holm-Sidak post-hoc test.

In contrast to tissue-specific upregulation of inflammatory cytokines, circulating serum cytokine levels are known to be only modestly impacted by HF diet feeding^36–38^. Cytometric bead array analyses on blood serum collected from the different treatment groups revealed equivalent serum cytokine levels for most markers tested (data not shown). One notable exception was the T cell regulatory chemokine C-X-C motif chemokine 10 (CXCL10)^39^, a biomarker for development of heart failure and adverse cardiac remodeling. CXCL10 was found to be upregulated in HF-diet fed mice, whereas (*R*)-DOI treated mice exhibited reduced levels that were similar to NC animals (Fig. 6).

## Discussion

The standard, ‘Western diet’ overloads blood with cholesterol, triglycerides, and glucose. This diet can result in altered glucose homeostasis, metabolic disease, and cardiovascular dysfunction. Traditional 3-hydroxy-3-methylglutaryl coenzyme A (HMG-CoA) reductase inhibitors, or statins, represent the gold-standard in lipid-lowering therapy due to their efficacy at reducing atherosclerotic plaques. However, muscular symptoms and/or elevation of hepatic aminotransferases during statin therapy are serious side effects that contribute to statin intolerance in a sizable population of patients^40^. For statin-intolerant patients, alternative therapies first start with dosage lowering or alternative dosing regimens. While these treatments can result in reduced myopathies and liver toxicity, their ability to provide a long-term reduction in cardiac risk has not been established. Furthermore, the use of non-statin lipid-lowering drugs constitutes an underutilized approach to hyperlipidemia. Preliminary results into the LDL-C reducing potential of various nutraceuticals and supplements is promising, but further study is necessary to confirm their effectiveness and safety^41^.

A recent study has found that cytokine-based/anti-inflammatory therapy may be more efficacious in preventing cardiovascular disease than cholesterol-lowering compounds^42^. Results from the CANTOS (Canakinumab Anti-Inflammatory Thrombosis Outcome Study) trial show that the IL-1β inhibitor canakunumab, independent of lipid-lowering, significantly reduces the risk of cardiovascular disease and recurrent cardiovascular events in patients with previously diagnosed myocardial infarction^43^. Statins themselves have been shown to possess a variety of anti-inflammatory properties, which has led to speculation that the inflammatory inhibiting effects of statins may be equally if not more important for their cardioprotective effects than simple cholesterol lowering^44^. Thus, when designing a drug to be an alternative to statins, any compound that lowers cholesterol levels while concurrently having anti-inflammatory properties is highly desirable.

We have previously shown that (*R*)-DOI has potent anti-inflammatory properties in primary vascular cells, against TNF-α-mediated inflammation in the aortic arch, and in a model of allergic asthma^45,24,46^. The levels of (*R*)-DOI necessary to produce these anti-inflammatory effects are low, and in some instances far below those necessary to produce behavioral effects. Frequent consumption of a “Western” diet is known to promote systemic inflammation and immune cell dysregulation^47^. HF-diet feeding has been shown by others to induce the expression of a number of proinflammatory factors that we have previously found to be elevated in our TNF-α and/or ovalbumin models (i.e. IL-5, IL-6, IL-13, MCP-1, GM-CSF)^48^; therefore, we examined the ability of (*R*)-DOI to protect against the effects of a high fat diet in the ApoE^−/−^ mouse model. We chose the ApoE^−/−^ model because this mouse robustly develops metabolic and vascular disease in response to being fed a “Western” style diet. As expected, we found that expression of proinflammatory genes, including the cell adhesion molecule *vcam*-1 and the cytokine *Il*-6, were significantly elevated in aortic tissues following 16 weeks of high-fat diet feeding (Fig. 5). Significantly, (*R*)-DOI prevented high fat diet-induced expression of several inflammatory markers, suggesting that 5-HT_2_ receptor activation is a viable strategy to combat diet-induced vascular inflammation. An interesting exception was for *tnf*-*α*, where the HF diet did not induce mRNA expression, but the combination of the HF diet and (*R*)-DOI resulted in significantly lowered *tnf-α* expression below that of even control. Another interesting result is the upregulation of the T-cell chemokine and cardiovascular disease biomarker CXCL10 in our HF-diet fed animals (Fig. 6). While the standard Western diet increased the overall expression of inflammatory markers in vascular tissues, its impact on circulating cytokines is minimal^36^. However, higher concentrations of CXCL10 (IP-10) have been found in the plasma of patients with coronary artery disease^49^, which has been theorized to modulate the balance of effector and regulatory T cells in atherogenesis^39^.

As mentioned above the 5-HT_2A_ receptor is present in cardiovascular tissue critical to autonomic functioning (vascular smooth muscle, endothelial cells, cardiomyoctyes) and the immune cell populations that resides in cardiac tissue (mononuclear phagocytes, neutrophils, B and T cells, macrophages)^13,50^. Accordingly, the receptor for CXCL10, CXCR3, is also expressed both in non-immune (endothelial and smooth muscle cells) and immune (T lymphoctyes, natural killer cells, monocytes) cardiovascular tissue^51^, with CXCL10 binding to CXCR3 mediating a plethora of cell functions, including chemotaxis, proliferation, migration and survival. As both 5-HT_2A_ and CXCR3 receptors reside on both these immune and non-immune cell populations, it’s possible a dynamic interplay exists between 5-HT_2A_ and CXCR3 receptor activation. Therefore, it is conceivable that the primary targets of (*R*)-DOI in this model are immune cells. The finding that elevated levels of CXCL10 induced by high fat feeding are reduced to control levels in mice administered (*R*)-DOI supports the notion that activation of 5-HT_2A_ receptors on discreet subsets of lymphocytes modulates vascular inflammation in a therapeutic manner. Further studies are necessary, however, to determine the role of (*R*)-DOI-mediated 5-HT_2_ receptor activation on these different cellular populations.

Impaired glucose tolerance is a major risk factor for adverse cardiovascular events^52,53^. In particular, population studies have revealed that impaired glucose metabolism combined with dyslipidemia are major risk factors for cardiovascular disease^54^. Here we demonstrated that (*R*)-DOI improved glucose tolerance for both HF and normal chow fed mice. We cannot yet say what the underlying mechanism of this effect is, but previous work by others has shown that beta-islet cells in the pancreas express 5-HT_2A_ receptors and that agonism of these receptors can increase insulin secretion^55^.

Interestingly, (*R*)-DOI decreased both total cholesterol and LDL cholesterol in HF-diet fed mice. The reduction in total and LDL cholesterol suggests a possible protective therapeutic effect for (*R*)-DOI against hypercholesterolemia; however, further experiments are necessary to fully understand the effects of this drug on cholesterol and how this may impact therapeutic strategies. Interestingly, cholesterol is known to directly interact with the 5-HT_2A_ receptor to induce increased receptor conformational variability^56^. One may speculate that conformational changes induced by cholesterol contribute to its anti-inflammatory effect by either enhancing interactions with anti-inflammatory signaling pathways or preventing interactions with downstream pro-inflammatory signaling cascades. This, however, will also require further experimentation. Polymorphisms at the *HTR2A* locus have been shown to be associated with cholesterol levels^57^. Therefore, 5-HT_2A_ receptor function in general may modulate other aspects of lipid homeostasis and that activation with (*R*)-DOI is affecting these processes.

Whereas (*R*)-DOI is an agonist of 5-HT_2_ receptors, previous work by others has demonstrated that antagonists for these receptors can protect against vascular inflammation. For example, the 5-HT_2_ receptor antagonist sarpogrelate retards the progression of atherosclerosis in rabbits^58^. We speculate that while the effects of (*R*)-DOI are active mediation of anti-inflammatory processes, 5-HT_2_ receptor antagonists may merely be blocking the well-established proinflammatory effects of serotonin. For example, 5-HT is known to have proliferative effects on vascular smooth muscle cells and macrophages. Sarpogrelate may simply be blocking the effects of 5-HT on these cells and preventing inflammation induced proliferation, resulting in protection against high fat diet-induced atherosclerosis and vascular inflammation. Based on our previous studies on the ability of (*R*)-DOI to prevent vascular-related cell and tissue inflammation induced by TNF-α, which is a key pro-inflammatory cytokine in atherosclerosis and vascular inflammation, via 5-HT_2A_ receptor activation we propose the following model. Sub-behavioral levels of systemic circulating (*R*)-DOI activate 5-HT_2A_ receptors to induce anti-inflammatory pathways that include blocking the expression of IL-6, VCAM-1, CXCL10, and TNF-α from vascular endothelial and smooth muscle cells as well as macrophages that ultimately limit high fat-induced vascular inflammation and recruitment of macrophages to the aorta that would otherwise differentiate to foam cells producing tissue damage and more inflammation. This reduced vascular inflammation may also include a component resulting from the observed decrease in total plasma and LDL cholesterol by drug treatment. Consistent with our proposed model, a recent study found that the antipsychotic drug olanzapine, a 5-HT_2A_ receptor inverse agonist, increases serum levels of total cholesterol, non-HDL, HDL-c, and triglycerides, deregulates hepatic lipid metabolism, and increases aortic proinflammatory protein expression (VCAM-1, TNF-α, and IL-6) in apoE^−/−^ mice^59^. Along a similar vein, the 5-HT_2C_ selective agonist lorcaserin effectively reduces appetite to induce weight loss, a result we do not see with (*R*)-DOI in our study. As lorcaserin is the first weight-loss drug proven to have cardiovascular safety^60^ and olanzapine worsens hyperlipidemia and aortic inflammation, our finding that (*R*)-DOI possesses vascular protective effects independent of weight-loss suggests that biased signaling at 5-HT_2_ receptors confers different therapeutic properties in vascular tissues. Further studies examining vascular plaques and lipid accumulation in the aorta and heart in disease models like the high fat diet-fed ApoE^−/−^ mouse will provide additional information regarding the potential of 5-HT_2_ receptor activation with sub-behavioral levels of (*R*)-DOI as a therapeutic strategy to treat cardiovascular disease and atherosclerosis.
